# Greener synthesized P-doped carbon for dual applications: selective cationic dye removal with phytotoxicity assessment and industrial effluent treatment

**DOI:** 10.1039/d6ra00482b

**Published:** 2026-03-31

**Authors:** M. Bhavani Lakshmi, Alibasha Akbar, Paramita Pattanayak, Tanmay Chatterjee, Archana V., Mihir Ghosh

**Affiliations:** a Department of Chemistry, SRM Institute of Science and Technology Kattankulathur 603203 Tamil Nadu India mihirg@srmist.edu.in; b Department of Chemistry, Birla Institute of Technology and Science, Pilani – Hyderabad Campus Jawahar Nagar, Kapra Mandal Hyderabad Telangana 500078 India; c CAMRIE, School of Physics, Indian Institute of Science Education and Research Thiruvananthapuram (IISER TVM) Maruthamala P.O., Vithura Thiruvananthapuram Kerala 695551 India

## Abstract

This study presents a novel approach to cationic dye remediation through the strategic valorization of onion peel (OP) waste—a precursor uniquely rich in organosulfur and flavonoid compounds into a phosphorus-doped activated carbon (OPP-1) *via* one-step H_3_PO_4_ activation. Unlike conventional lignocellulosic wastes, the inherent heteroatom content and biochemical complexity of OP synergize with phosphoric acid to produce a mesoporous carbon (535.5 m^2^ g^−1^) with enhanced surface functionality, hydrophilicity (contact angle: 10.1°), and strong negative surface charge (−14.4 mV). OPP-1 exhibits exceptional selectivity for cationic dyes, achieving >90% removal of safranin-O (99.99%), methylene blue, brilliant green, and methyl violet, with significantly lower uptake of anionic dyes. Safranin-O adsorption followed Langmuir and pseudo-second-order kinetics, indicative of monolayer chemisorption driven by electrostatic, π–π, and hydrogen-bonding interactions. The synthesized nanocomposite was tested using two textile effluents (IW-1 and IW-2) by time-dependent UV-visible spectroscopy. Dye adsorption was confirmed by a constant decrease in absorbance at 512 and 510 nm. It was able to remove 95.80% of IW-1 within 50 minutes and 98.75% of IW-2 within 60 minutes. Notably, phytotoxicity assays using *Vigna radiata* demonstrated substantial detoxification, with root and shoot growth recovering to 91% and 82% of control levels, respectively. The adsorbent also maintained 58% efficiency over 10 regeneration cycles. This work highlights a sustainable, waste-to-resource strategy for designing highly selective adsorbents, positioning OP-derived phosphorous-doped carbon as an effective and eco-friendly solution for cationic dye contamination.

## Abstract 2

Phosphorus-doped activated carbon (OPP-1) derived from onion peel *via* one-step H_3_PO_4_ activation enables efficient cationic dye adsorption.

## Graphical abstract

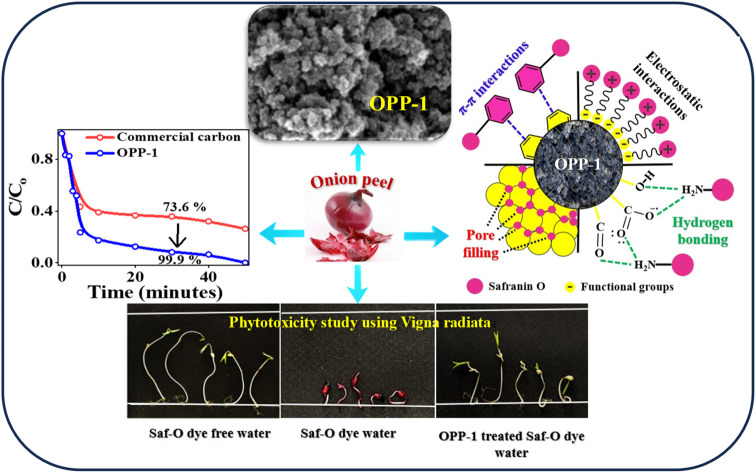

## 1. Introduction

The treatment of industrial wastewater containing persistent synthetic dyes remains a significant environmental challenge, with cationic dyes posing particular concern due to their high toxicity, stability, and resistance to conventional degradation processes.^1^ Among the various remediation strategies, adsorption using carbonaceous materials is considered one of the most effective approaches owing to its operational simplicity, broad applicability, and potential for regeneration. However, many reported bio-derived activated carbons exhibit limited selectivity toward cationic species, moderate adsorption capacities, or rely on post-synthetic chemical functionalization to introduce surface charge and active binding sites, thereby increasing process complexity and environmental burden. A fundamental limitation in current biomass-derived carbons lies in precursor selection. Most commonly used agricultural wastes, such as rice husk,^2^ sugarcane bagasse,^3^ sawdust,^4^ and coconut shell,^5^ are lignocellulosic in nature. Direct carbonization yields carbons with chemically inert basal planes and limited porosity, necessitating activation (chemical or physical) to develop a high-surface-area porous structure and introduce surface functional groups for effective dye adsorption. Furthermore, precursor-specific challenges exist: silica-rich precursors (*e.g.*, rice husk) require demineralization to prevent pore blockage,^6^ while highly lignified precursors (*e.g.*, coconut shell) typically yield rigid, microporous-dominated carbons that can limit the intra-particle diffusion of bulky dye molecules.^7^

In this context, OP (*Allium cepa* L.) represents a distinctly advantageous biomass precursor, intrinsically predisposed to yield functionally active carbons.^8,9^ Unlike rice husk, coconut shell, which yields chemically inert basal planes requiring extensive post-synthetic modifications, OP possesses a unique biochemical profile enriched with flavonoid glycosides (notably quercetin derivatives), organosulfur compounds (*S*-alkenyl cysteine sulfoxides), and abundant oxygenated functionalities,^10^ which provides inherent aromatic precursor and embedded heteroatoms (O, S, N). These elements are retained and reconfigured within the carbon matrix during thermal treatment, circumventing the need for secondary doping. Furthermore, OP's thin, lamellar morphology and low inorganic ash content facilitate uniform chemical penetration and minimize pore obstruction during activation. Phosphorus doping *via* one-step H_3_PO_4_ activation enhances cationic dye removal through dual mechanisms: (i) H_3_PO_4_ promotes crosslinking and dehydration reactions within the biomass matrix, generating phosphorus-containing functional groups that increase surface acidity and negative charge, and (ii) it simultaneously develops well-defined porosity.^11,12^ This process concurrently results in the *in situ* incorporation of phosphorus alongside the native heteroatoms, generating a co-doped (O–S–P) carbon surface rich in acidic oxygen and phosphorus groups.^13,14^ This imparts a strongly negative surface charge, ideal for the selective adsorption of cationic dyes *via* a combination of dominant electrostatic attraction, supplemented by hydrogen bonding and π–π interactions with condensed aromatic domains. Importantly, this precursor-driven functionalization strategy eliminates the need for external dopants, post-synthetic surface modification, or metal-based activators, thereby aligning with green chemistry and circular-economy principles. By rationally exploiting the inherent molecular and structural attributes of OP, it becomes possible to achieve adsorption performance metrics comparable to those of synthetically engineered carbons, while maintaining process simplicity and sustainability. Furthermore, the abundant availability of onion peel as agricultural waste, combined with the straightforward, single-step H_3_PO_4_ activation process at a moderate temperature of 600 °C, makes the production of OPP-1 highly feasible for scale-up and bulk manufacturing for practical wastewater treatment applications. To assess the environmental safety of the treated effluent and confirm the detoxification capability of the adsorbent, phytotoxicity studies were conducted using *Vigna radiata* (mung bean). Its rapid germination, sensitivity to pollutants, and established role as a model organism in ecotoxicological studies, coupled with its agricultural importance (high nutritional value, low water requirement), make it suitable for evaluating the potential reuse of treated wastewater in irrigation. Monitoring its germination and growth in dye-contaminated *versus* OPP-1-treated water thus provides a reliable indicator of detoxification and environmental compatibility.^15^

Herein, we report the one-step synthesis of a phosphorus-doped activated carbon (denoted OPP-1) derived from OP *via* H_3_PO_4_ activation and evaluate its performance for the selective removal of cationic dyes, using safranin-O as a model pollutant. Comprehensive multiscale characterization (XRD, XPS, FTIR, Raman spectroscopy, BET, SEM, TEM, and zeta potential analysis) is employed to establish direct correlations between precursor-derived heteroatoms, phosphorus doping, pore architecture, and surface charge. Adsorption behavior is systematically investigated through kinetic and isotherm models, selectivity studies, and regeneration experiments. The performance of OPP-1 is further validated using actual textile industrial wastewater samples to show practical applicability. Without the use of external oxidizing or reducing agents, OPP-1 achieves rapid removal efficiencies exceeding 95%. Beyond adsorption efficiency, the environmental relevance of the treatment is assessed through phytotoxicity bioassays using *Vigna radiata*, demonstrating effective detoxification of dye-contaminated water. This study highlights a rational precursor-selection paradigm for designing high-performance, waste-derived adsorbents with built-in functionality for sustainable wastewater remediation.

## 2. Experimental section

### 2.1. Materials

Activated carbon is made from OP (*Allium cepa*) that have been collected from agricultural fields through local farmers in Sennikulam village, Tenkasi district. Hydrochloric acid (HCl, SRL Chemicals, India), sodium hydroxide (SRL Chemicals, India), ethanol (SRL Chemicals, ≥85%, India), orthophosphoric acid (SRL Chemicals, India), potassium dihydrogen orthophosphate (SRL Chemicals, India), anhydrous potassium phosphate dibasic (SRL Chemicals, ≥99.5%, India), and Safranine-O (Saf-O; SRL Chemicals, India) were used directly without additional purification. 1000 mg of each dye were dissolved in 1 L of deionized water to create stock dye solutions (1000 mg L^−1^), which were then diluted as necessary. Phosphate buffer (KH_2_PO_4_/K_2_HPO_4_) with 0.1 M NaOH or HCl was used to adjust the pH.

### 2.2. Synthesis of OP-derived activated carbon

**Fig. 1:**
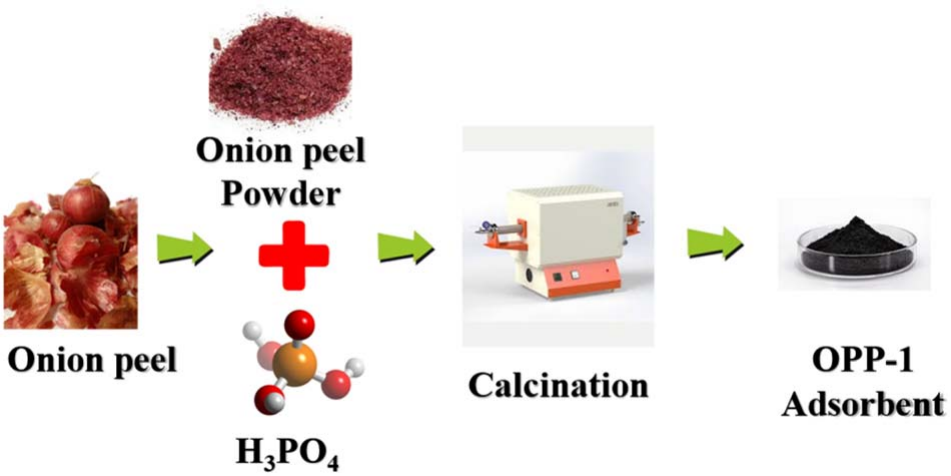
Preparation of activated carbon derived from OP using orthophosphoric acid activation.

OPs were collected from the agricultural land was thoroughly washed with deionized water, and oven-dried at 50 °C for 12 hours. The dried biomass was ground, sieved for uniformity, and stored in airtight containers. Chemical activation used orthophosphoric acid (H_3_PO_4_) at a 1 : 4 (w/v) OP to acid ratio, with impregnation for 1–48 hours at ambient temperature. Impregnated samples were carbonized in a nitrogen atmosphere by heating to 600 °C at a rate of 10 °C min^−1^ and holding for 1 hour. The resulting carbon was repeatedly washed with distilled water to neutral pH, dried at 105 °C overnight, and stored for characterization and adsorption studies. The synthesis scheme is shown in Fig. 1.

### 2.3. Characterizations

XRD patterns were obtained using a Panalytical Xpert Pro diffractometer (Cu Kα, 0.154 nm; 2*θ* = 10–80°, 0.025° steps). FTIR spectra (4000–400 cm^−1^) were recorded with a Bruker Alpha T in ATR mode. Surface morphology and elemental composition were analyzed by FESEM-EDS (FEI Apreo S) and HRTEM (JEOL 2010F, 200 kV). BET and BJH analyses (N_2_ adsorption–desorption) were performed with a Microtrac BEL SORP mini II. Atomic force microscopy (AFM) with a JPK-NANOWIZARD 4 instrument was used to study surface characteristics. XPS measurements employed a PHI Versa Probe III (Al Kα). Raman spectra were collected with a HORIBA LabRam HR Evolution (532 nm). Particle size and zeta potential were measured by dynamic light scattering (Malvern ZS-3600). UV-vis spectra (Agilent Cary 60), solid-addition pH_PZC_, and Boehm titration completed the materials' characterization.

### 2.4. Batch adsorption experiments

Batch adsorption experiments of dye onto OPP-1 were conducted under different experimental conditions to identify the optimum adsorption parameters. The adsorbent dose, pH, contact time, initial dye concentration, and solution temperature were all varied to examine the adsorption of dye molecules from an aqueous solution onto OPP-1. Following every experiment, a Büchner funnel was used to filter the dye solutions through Whatman filter paper grade 40. A UV-vis spectrophotometer was used to measure the amount of dye that was still present in the filtrate at each dye's corresponding maximum wavelength (*λ*_max_). To determine the % removal efficiency, the following formula was used:1
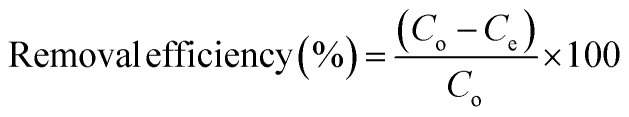
where *C*_o_ and *C*_e_ (mg L^−1^) are dye concentrations at the initial and equilibrium, respectively.

The effect of adsorbent dosage (10–100 mg), pH (2–12), contact time (0–50 min), initial dye concentration (10–100 mg L^−1^), and the effect of temperature (30–80 °C) were considered. The equilibrium adsorption capacity (*q*_e_, mg g^−1^) was calculated using the following equation:2
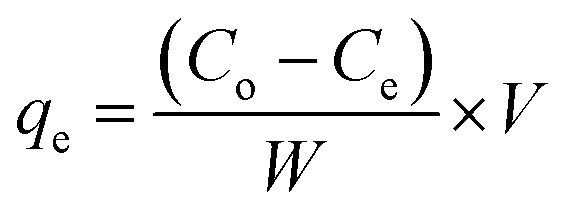


The volume of the solution and the mass of the solid adsorbents are represented here as *V* (L) and *W* (g), respectively.

### 2.5. Results and discussions

**Fig. 2:**
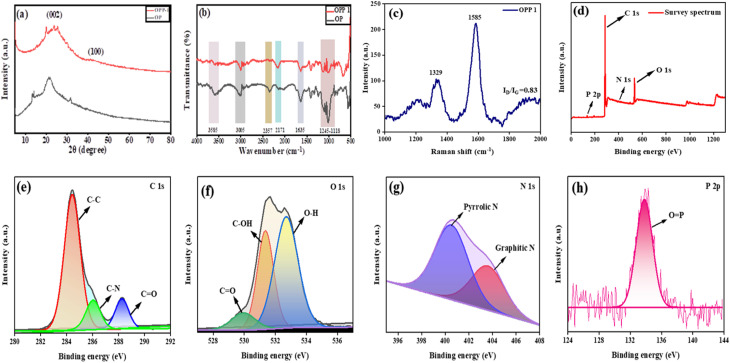
(a) XRD pattern of OP and OPP-1 and (b) FTIR spectrum of OP and OPP-1 (c) Raman spectrum of OPP-1, (d–h) XPS spectra of OPP-1 activated carbon: (d) survey spectra (e) C 1s core level spectra (f) O 1s core level spectra (g) N 1s core level spectra, and (h) P 2p core level spectra.

XRD analysis of OP and the activated carbon OPP-1 (Fig. 2a) reveals a predominantly amorphous structure, with a broad diffuse peak at 2*θ* ≈ 23°, corresponding to the (002) plane of disordered graphitic layers.^15–17^ A weaker broad signal near 2*θ* ≈ 43° aligns with the turbostratic (100) plane, further indicating limited graphitic stacking. The broad and low-intensity peaks confirm low crystallinity and minimal graphitization, typical of biomass-derived carbons subjected to phosphoric acid activation.^18^ This amorphization results from dehydration and glycosidic linkage cleavage during thermal activation, leading to the collapse of crystalline cellulose domains (specifically absent peaks between 14–22°). The resulting disordered carbon matrix exhibits abundant surface defects and microporosity, which optimize active sites and accessibility for adsorption processes. Such structural features are advantageous in enhancing pollutant capture and diffusion in water treatment applications. The XRD pattern of recycled OPP-1 demonstrates large peaks at 2*θ* = 23° and 43°, which correspond to the disordered carbon's (002) and (100) planes. This confirms that the amorphous structure of the material has been retained after regeneration (Fig. S1a). The FTIR spectrum of OP and OPP-1 (Fig. 2b) shows characteristic surface groups introduced during activation. A broad band at 3585 cm^−1^ corresponds to O–H stretching of hydroxyl groups (phenolic/alcoholic), retained due to incomplete dehydration.^15,19^ The peak at 3000 cm^−1^ indicates aliphatic C–H stretching, while the weak band at 2357 cm^−1^ arises from adsorbed atmospheric CO_2_. The 2171 cm^−1^ band is attributed to C≡C or C≡N triple bonds formed during pyrolysis.^20,21^ The strong absorption at 1647 cm^−1^ corresponds to C=C stretching in aromatics or C=O in conjugated carbonyls. Peaks between 1245 and 1118 cm^−1^ are due to P–O–H, O–C (P–O–C), and P–OOH groups from phosphoric acid treatment. The 1068 cm^−1^ band relates to symmetric vibrations of ionized P–O in phosphate esters and P–O–P chains.^22–25^ The characteristic O–H (∼3585 cm^−1^), C=C/C=O (∼1647 cm^−1^), and phosphorus-containing functional group bands (1245–1068 cm^−1^) are still present in the recycled OPP-1's FTIR spectra, suggesting that surface chemistry is preserved throughout regeneration (Fig. S1b). Raman spectroscopy of OPP-1 (Fig. 2c) displayed two prominent bands at 1329 cm^−1^ (D band) and 1584 cm^−1^ (G band), corresponding to the A_1g_ breathing mode of disordered carbon (sp^3^ hybridized) and the E_2g_ stretching vibration of sp^2^-bonded graphitic carbon, respectively. The measured *I*_D_/*I*_G_ ratio of 0.83 signifies that OPP-1 comprises nanocrystalline graphitic domains dispersed within an amorphous carbon matrix.^18^ This ratio reflects a substantial degree of structural disorder and a reduced in-plane crystallite size, characteristic of partially ordered carbon materials. The coexistence of disordered and graphitic regions imparts a defect-enriched surface architecture, which is expected to facilitate enhanced adsorption with Saf-O dye molecules.^26,27^

**Fig. 3:**
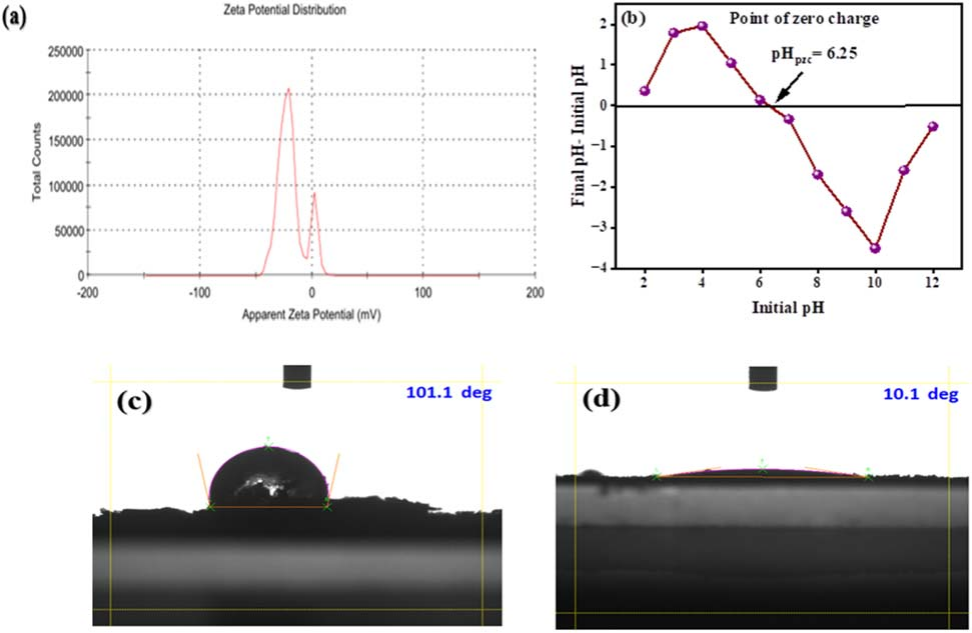
(a) Zeta potential measurement of OPP-1, (b) determination of the point of zero charge of OPP-1, contact angle measurement of (c) OP, and (d) OPP-1.

The XPS spectra of the H_3_PO_4_-activated carbon confirmed the coexistence of carbon, oxygen, nitrogen, and phosphorus elements on the surface (Fig. 2d–h). The high-resolution C 1s spectrum exhibited three distinct peaks at 284.48, 286.08, and 288.28 eV, corresponding to C–C, C–N, and C=O functional groups, respectively.^28,29^ The O 1s spectrum was deconvoluted into three components centered at 530.08, 531.38, and 532.68 eV, which can be ascribed to lattice oxygen (C=O), hydroxyl oxygen (C–OH), and adsorbed oxygen (O–H) species, respectively.^30^ The N 1s spectrum revealed two peaks at 400.58 and 403.68 eV, attributed to pyrrolic N and graphitic N, confirming the successful incorporation of nitrogen functionalities into the carbon framework.^29^ Additionally, the P 2p spectrum displayed a distinct peak at 133.8 eV, characteristic of P=O species derived from phosphoric acid activation.^31,32^ The presence of these oxygen-, nitrogen-, and phosphorus-containing groups, in agreement with the FTIR results, indicates that H_3_PO_4_ activation not only improves the surface porosity but also introduces diverse heteroatom functionalities that enhance the surface reactivity and adsorption performance of the activated carbon. Dynamic light scattering (DLS) analysis of the synthesized OPP-1 revealed an average hydrodynamic diameter of 2802 nm with a polydispersity index (PDI) of 0.298, indicating a moderately uniform particle size distribution, as shown in Fig. S1c. The PDI range (0–1) reflects dispersion uniformity, where lower values correspond to narrower size distributions and improved colloidal stability. Smaller particle sizes generally correlate with higher surface areas, beneficial for adsorption applications.^33–35^ Zeta potential measurements (Fig. 3a) showed a value of −14.4 mV, indicating a net negative surface charge on OPP-1 particles. This negative charge promotes adsorption of cationic dyes *via* electrostatic attraction between the positively charged dye molecules and negatively charged surface functional groups, such as ionized hydroxyl and oxygen-containing moieties.^36^ The point of zero charge (pH_PZC_) of the OPP sample was determined using the solid addition method. The plot of ΔpH = (pH_f_ − pH_i_) *versus* initial pH (pH_i_) indicated that the pH_PZC_ occurred at 6.25 (Fig. 3b), where the net surface charge is neutral. Below this pH, the surface is positively charged, while above it, the surface acquires a negative charge. At pH values above 6.25, the negatively charged OPP-1 surface enhances adsorption of the cationic dye Saf-O through increased electrostatic attraction between the dye molecules and ionized surface functional groups.^37,38^

The surface functional groups of OPP-1 were quantitatively evaluated using Boehm titration to better understand its adsorption behavior.^39,40^ OPP-1 exhibited a predominance of acidic sites, mostly carboxylic groups (0.95 mmol g^−1^), with a smaller amount of lactonic (0.01 mmol g^−1^) and phenolic (0.04 mmol g^−1^) functionalities (Table S1). Through π–π interactions, hydrogen bonds, and electrostatic attraction, these oxygen-containing groups promote cationic dye adsorption. The presence of hydroxyl, carbonyl, and phosphorus-containing functional groups on the OPP-1 surface is confirmed by FTIR analysis, which displays a broad O–H stretching band at 3585 cm^−1^, a C=O/C=C band at 1647 cm^−1^, and particular phosphate-related vibrations in the 1245–1118 and 1068 cm^−1^ regions.

Surface wettability significantly influences the effectiveness of adsorbents in removing dyes from water. Contact angle (*θ*) measurements categorize surfaces as super-hydrophilic (*θ* < 10°), hydrophilic (10° < *θ* < 90°), hydrophobic (90° < *θ* < 150°), or super-hydrophobic (*θ* > 150°), with lower angles indicating higher polarity and water affinity.^41,42^ Drop contour analysis showed that raw OP had a contact angle of 101.0°, indicating hydrophobicity (Fig. 3c). In contrast, phosphoric acid-activated carbon (OPP-1) exhibited a substantially lower contact angle of 10.1°, classifying it as hydrophilic (Fig. 3d).^43,44^ The increased wettability results from the enrichment of oxygen-containing surface groups through chemical activation, as confirmed by FTIR. This hydrophilic property improves water compatibility and facilitates efficient dye adsorption.^45^

**Fig. 4:**
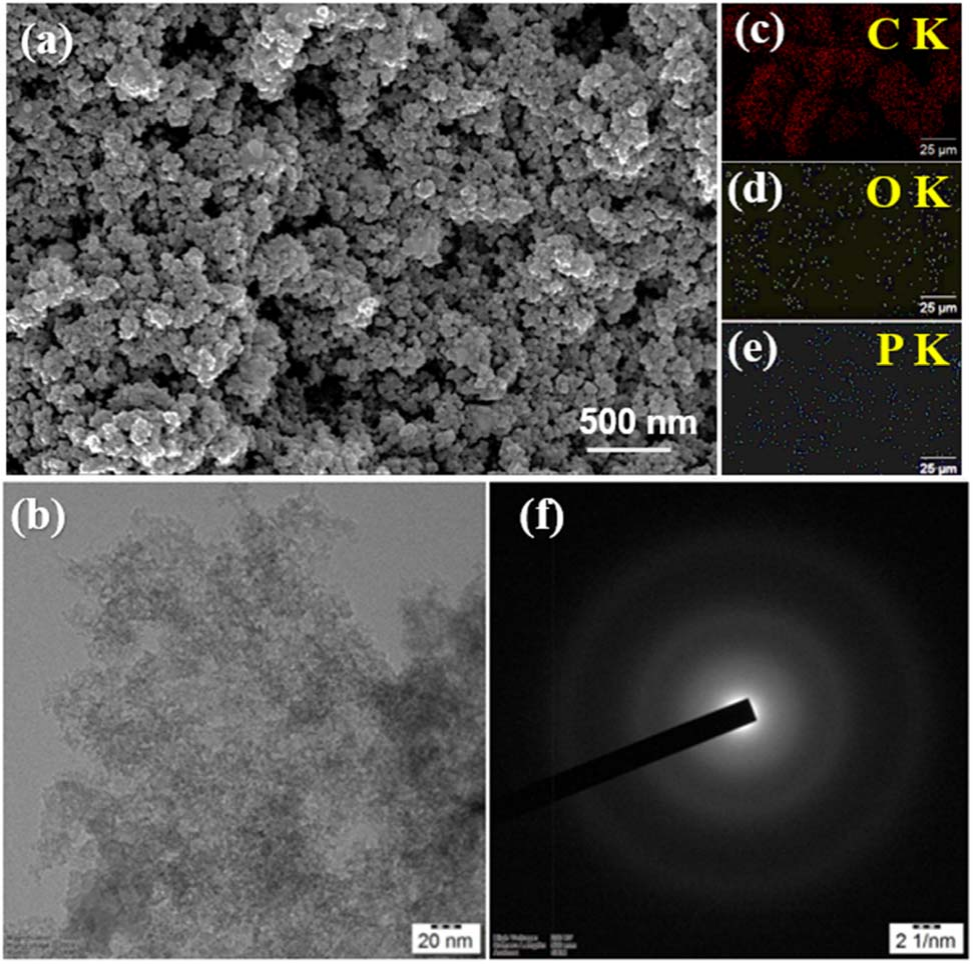
(a) SEM images of OPP-1, (b) TEM image of OPP-1, and elemental mapping of (c) carbon, (d) oxygen, (e) phosphorus, (f) SAED pattern of OPP-1.

SEM analysis of OPP-1 activated carbon (Fig. 4a) reveals a highly porous and irregular surface morphology with interconnected aggregates and numerous mesopores distributed throughout the matrix. These features indicate successful phosphoric acid activation, which facilitates extensive fragmentation of the biomass precursor and formation of a three-dimensional porous network. Higher magnifications highlight pore-like cavities and open channels, further evidencing the collapse of cellulose domains and the generation of a sponge-like texture optimal for adsorption. Elemental mapping (Fig. 4c–e) further complements the morphological observations by confirming the elemental distribution and chemical homogeneity of the OPP-1 sample. EDX analysis detects predominant signals for C, O, and P consistent with phosphorus incorporation following activation. The corresponding elemental maps show that carbon, oxygen, and phosphorus are uniformly distributed across the carbon matrix, indicating effective doping and functionalization during synthesis. These structural and textural properties, including the presence of functional groups such as –OH, P–O–H, O–C (P–O–C), P–OOH, and P–O–P, substantially enhance water compatibility and facilitate rapid adsorption of pollutant molecules, confirming the suitability of OPP-1 for aqueous dye removal and environmental remediation applications. To further examine the surface features, AFM analysis was performed, as shown in Fig. S2. In the AFM image, distinct peaks and hills are observed across the surface, indicating nanoscale topographical variations. TEM provided high-resolution insight into the nanoscale structural organization of OPP-1 activated carbon in Fig. 4b. Low-magnification TEM images revealed a highly disordered, sponge-like arrangement of carbon domains with loosely packed aggregates and internal voids^46^ (Fig. S3a). As the magnification increased, the images exposed the presence of mesoporous channels distributed throughout the network, confirming the material's porosity. High-magnification (Fig. S3b) and lattice-resolved TEM visualized a predominantly amorphous structure, further supported by the diffuse ring pattern in selected area electron diffraction (SAED) in Fig. 4f, indicating short-range order interspersed within the carbon matrix. These nanoscale TEM observations directly corroborate the morphological features seen in the SEM, which revealed a rough, porous surface architecture with abundant mesopores. While SEM characterizes the exterior topology and mesostructural connectivity, TEM demonstrates that both the external surface and internal framework are permeated by pore channels and disordered carbon regions. This interlinked, multi-scale pore network extending from nanometer to micrometre dimensions reflects the efficiency of phosphoric acid activation in creating a high surface area, defect-rich material optimal for adsorption in aqueous environments.

**Fig. 5:**
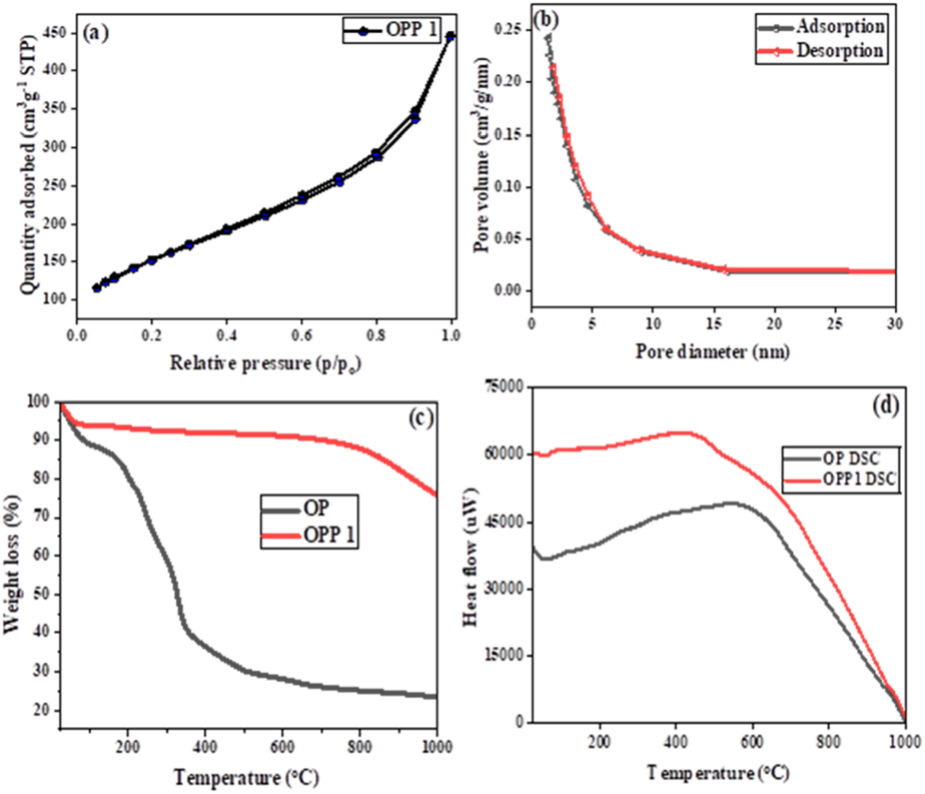
(a) BET isotherm of OPP-1 nanocomposite and (b) BJH plot, (c) TGA spectrum of OP and OPP-1, and (d) DSC spectrum of OP and OPP-1.

Textural properties of the synthesized OPP-1 sample were assessed *via* N_2_ adsorption–desorption isotherms at 77 K (Fig. 5a). The isotherm exhibits a Type IV(a) profile with an H3 hysteresis loop, characteristic of mesoporous materials with slit-shaped pores formed by particle aggregation.^47,48^ These findings align well with morphological observations from SEM and TEM. BET analysis indicates a high specific surface area of 535.5 m^2^ g^−1^ and total pore volume of 0.69 cm^3^ g^−1^. The Barrett–Joyner–Halenda (BJH) pore size distribution in Fig. 5b confirms dominant mesoporosity centered at 3–4 nm, highlighting OPP-1's suitability for efficient adsorption in aqueous environments.

Thermogravimetric analysis (TGA) of raw OP exhibited three distinct degradation stages (Fig. 5c). Initial weight loss below 150 °C was attributed to the evaporation of moisture and the release of volatiles. A major decomposition phase between 150–350 °C corresponded to the breakdown of hemicellulose and cellulose, followed by the degradation of lignin and residual organics up to 600 °C, leaving a minimal char residue at higher temperatures.^18,49^ During the activation process, phosphoric acid (H_3_PO_4_) undergoes thermal decomposition, producing phosphorus pentoxide (P_2_O_5_) and water. The release of water vapor promotes pore formation through carbon gasification, contributing to the development of a porous carbon structure. The reaction can be represented as:

3 C + H_2_O → CO + H_2_

In addition, the P_2_O_5_ generated during the decomposition of H_3_PO_4_ can act as an oxidizing agent at elevated temperatures, which further promotes the etching of the carbon framework and enhances pore formation. This reaction can be expressed as:

4 P_2_O_5_ + 5C → 2P + 5CO

These reactions contribute to the formation of a highly porous carbon structure with increased surface area and active sites, which are beneficial for the adsorption of dye molecules from aqueous solutions.^50^

In contrast, phosphoric acid-activated carbon (OPP-1) showed markedly improved thermal stability, with only slight weight loss up to 600 °C and a gradual decline thereafter, retaining substantial residue even at 1000 °C. This enhanced stability supports the selection of 600 °C as the optimal pyrolysis temperature, balancing complete decomposition of labile components while preserving char yield and structural integrity.

Differential scanning calorimetry (DSC) profiles of OP and OPP-1 (Fig. 5d) exhibit exothermic peaks confirming combustion events. The broad exothermic region between approximately 170° and 850 °C relates to the thermal degradation of cellulose and hemicellulose. Notably, OPP-1 released higher total heat than OP, reflecting its improved thermal resistance and more intense combustion behaviour.^51^ The pronounced thermal transitions around 600 °C align with changes in carbonisation crystallinity, further justifying the choice of pyrolysis temperature for subsequent synthesis under inert conditions.

## 3. Adsorption behaviour of the prepared adsorbent

**Fig. 6:**
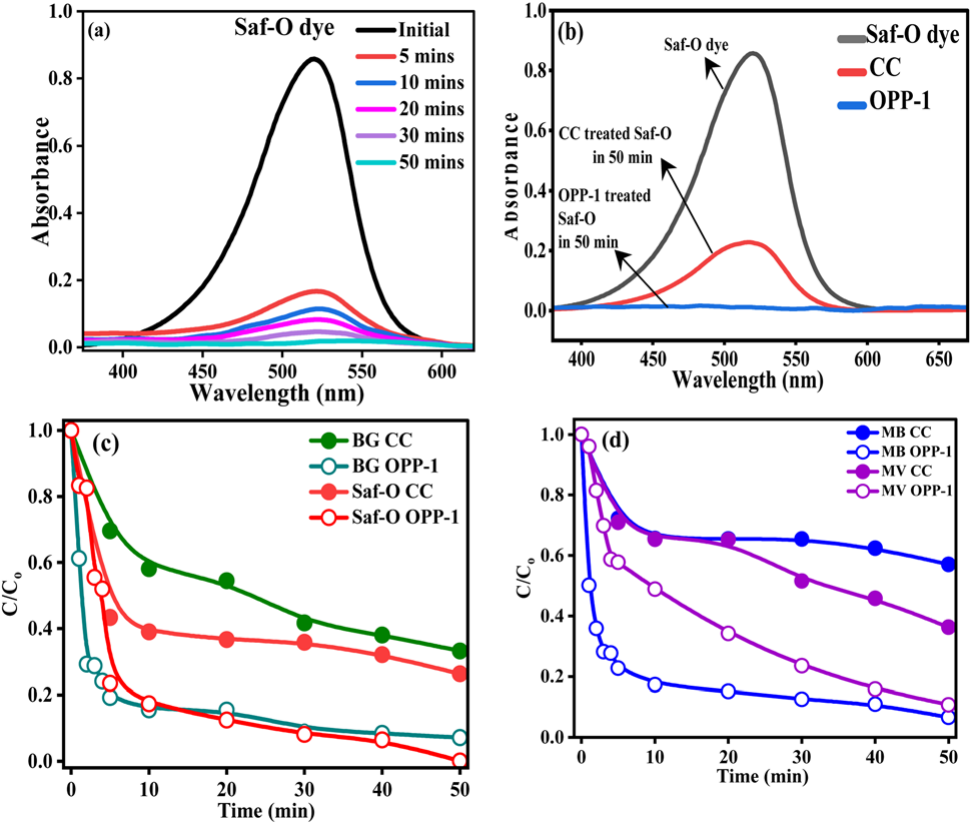
(a) UV-vis spectra depicting the adsorption of Saf-O dye in the presence of OPP-1, (b) UV-visible spectra of Saf-O after treatment with CC and OPP-1 in 50 minutes, (c) time-dependent adsorption of Saf-O and BG dyes on CC and the OPP-1 adsorbent (d) time-dependent adsorption of MB and MV dyes on CC and the OPP-1 adsorbent, illustrating comparative uptake efficiencies over time.

The above-discussed characterizations confirmed the presence of diverse surface functional groups on the synthesized adsorbents, enabling interactions such as intra-particle diffusion, complexation, hydrophobic and π–π interactions, pore filling, hydrogen bonding, and electrostatic attraction with pollutants. To evaluate adsorption performance, the OPP-1 sample was tested using Saf-O dye. UV-visible spectroscopy monitored dye removal at equilibrium, showing a pronounced decrease in absorbance at the dye's maximum wavelength (*λ*_max_ ≈ 520 nm, Fig. 6a). The OPP-1 sample demonstrated the highest removal efficiency, achieving significant dye uptake within 50 minutes. These results indicate the excellent efficacy of OPP-1 as an adsorbent for Saf-O in aqueous systems.

The adsorption performance for Saf-O has been measured using commercial activated carbon (CC) and the synthesized OPP-1 adsorbent (Fig. 6b). The untreated dye solution exhibited its characteristic absorption bands, which were only slightly decreased after treatment with commercial activated carbon, indicating minimal dye adsorption. On the other hand, OPP-1 resulted in a total elimination of the Saf-O peaks, with absorbance values close to the baseline. The measured spectral decline suggests a significantly greater adsorption efficiency of OPP-1 compared to commercial activated carbon, highlighting its potential for effective removal of cationic dyes.

The time-dependent adsorption behavior of BG and Saf-O on CC and the synthesized OPP-1 adsorbent is illustrated in Fig. 6c, and the MB and MV dyes are shown in Fig. 6d. OPP-1 exhibits a significant reduction in *C*/*C*_0_ over the measured time period for all dyes, indicating its superior dye affinity and enhanced surface interactions. In contrast, CC exhibits a gradual decrease in *C*/*C*_0_, suggesting comparatively weaker adsorption. OPP-1 constantly shows lower *C*/*C*_0_ values compared to CC across all dye systems, indicating its superior adsorption performance. These findings demonstrate OPP-1's improved structural properties and surface functionality, which promote more efficient dye removal.

### 3.1. Adsorption parameters

#### 3.1.1 Effect of pH

**Fig. 7:**
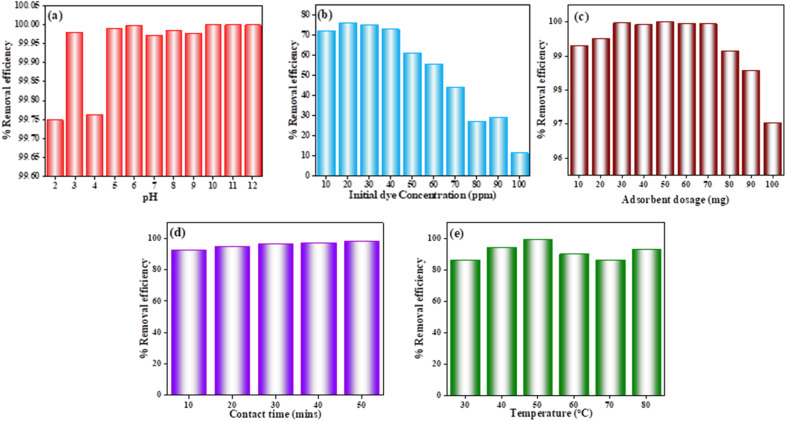
(a) Effect of pH, (b) effect of initial dye concentration, (c) effect of adsorbent dosage, (d) effect of contact time, and (e) effect of temperature.

The influence of solution pH on the adsorption efficiency of OPP-1 toward Saf-O dye was evaluated over a pH range of 2–12 (Fig. 7a). The adsorbent exhibited consistently high removal efficiencies exceeding 99.75% across this entire range, demonstrating excellent chemical stability and adaptability for diverse wastewater conditions. A slight reduction in removal efficiency at highly acidic pH 2 (99.75%) is attributed to protonation of surface groups and competition between excess H^+^ ions and dye molecules for adsorption sites, which marginally inhibits uptake. Removal efficiency notably improved at pH 3 (99.98%) and remained stable (99.76%) at pH 4. From pH 5 onward, adsorption efficiency was essentially constant, ranging between 99.97% and 99.99%, with the highest observed at pH 10 (99.99%). Enhanced performance in neutral to alkaline media is explained by deprotonation of functional groups such as hydroxyl and carboxyl moieties, which increases negative surface charge and strengthens electrostatic attraction to the cationic dye.^52^ The broad pH stability of OPP-1 underscores its potential for real-world applications, where influent pH variability is common.

#### 3.1.2 Effect of initial dye concentration

The influence of initial dye concentration (IDC) on the adsorption performance of OPP-1 was evaluated over 10–100 ppm (Fig. 7b). At low concentrations (10–30 ppm), removal efficiencies were exceptionally high (>99.5%), with optimum uptake at 20 ppm (99.94%). This enhanced efficiency arises because the adsorbent's abundant active sites exceed the number of dye molecules, enabling near-complete adsorption, consistent with similar observations in mesoporous adsorbents. As IDC increased beyond 30 ppm, removal efficiency progressively decreased, dropping to 96.33% and 95.88% at 40 and 50 ppm, and falling further at higher concentrations (*e.g.*, 68.82% at 80 ppm, 58.37% at 100 ppm). The significant reduction is attributed to site saturation: dye molecules increasingly compete for the limited available adsorption sites, which diminishes adsorption capacity. An anomalous efficiency rise at 90 ppm (90.08%) is likely due to experimental variability or transient dye redistribution effects, as reported in other studies.^53,54^ This concentration-dependent behavior underscores a fundamental adsorption principle where, at low IDC, surface sites are plentiful relative to adsorbate, facilitating efficient adsorption, while at high IDC, site limitation governs capacity reduction. Such effects highlight the critical need for equilibrium isotherm analyses to understand and optimize adsorbent utilization for large-scale wastewater treatment applications.

#### 3.1.3 Effect of adsorbent dosage

The impact of adsorbent dose on Saf-O removal efficiency was evaluated over a range of 10–100 mg (Fig. 7c). Removal efficiency increased with dosage, reaching 99.29% at 10 mg and peaking at 99.98% at 50 mg, attributable to the greater availability of active sites and surface area enhancing dye-adsorbent interactions. Between 30 and 70 mg, removal remained consistently above 99.9%, indicating equilibrium and near-complete dye adsorption from solution. However, beyond 70 mg, efficiency slightly declined to 99.14%, 98.57%, and 97.03% at 80, 90, and 100 mg, respectively, likely due to particle aggregation reducing effective surface area and site overlap, as well as insufficient dye molecules to saturate the excess adsorbent.^55^ Overall, 50 mg represents an optimal dosage balancing performance and material use, critical for scaling adsorption processes.

#### 3.1.4 Effect of contact time

Removal efficiency increased sharply from 89.81% at 5 min to 99% at 50 min (Fig. 7d), reflecting the rapid initial adsorption driven by the abundance of surface sites and efficient external mass transfer. The adsorption rate slowed after 20 min as active sites became progressively occupied, and the driving concentration gradient decreased. Near-equilibrium was approached around 30 min, with maximal dye removal achieved by 50 min, establishing this duration as sufficient for practical adsorption processes.^56^

#### 3.1.5 Effect of temperature

Temperature significantly influenced adsorption, with removal efficiency increasing from 86.07% at 30 °C to a maximum of 99.11% at 50 °C, indicative of an endothermic adsorption mechanism (Fig. 7e). Elevated temperatures enhance adsorbate mobility and potentially activate additional adsorption sites, thus improving dye uptake. Above 50 °C, efficiency declined to 89.69% at 60 °C and 85.92% at 70 °C, potentially due to desorption effects and thermal disruption of adsorbent–adsorbate interactions, before partially recovering to 92.79% at 80 °C. These results highlight the importance of temperature optimization for maximizing adsorption efficacy.^57^

## 4. Adsorption isotherm

Adsorption isotherms characterize the equilibrium distribution of adsorbate between liquid and solid phases at constant temperature and pH, providing insight into the adsorbent–adsorbate interaction mechanisms.^58^ In this study, the Langmuir, Freundlich, and Dubinin–Radushkevich isotherm models were applied to analyze the adsorption of Saf-O onto OPP-1 adsorbents. The Langmuir isotherm model provided the best fit to the experimental data, whereas the Freundlich and Dubinin–Radushkevich models are presented in the SI in Fig. S4a and b, and the values are given in Table 1.

**Table 1:** Adsorption isotherm models of OPP-1

Equilibrium model	Parameter	Saf-O
Langmuir isotherm	*q*_m_ (mg g^−1^)	40.978
	*K*_L_ (L mg^−1^)	1.124
	*R* ^2^	0.975
Freundlich isotherm	*K*_f_ (mg g^−1^)	262.670
	*n*	5.701
	*R* ^2^	0.692
Dubinin–Radushkevich isotherm	*K*_d_ (kJ^2^ mol^−2^)	−5.504
	*E* (kJ mol^−1^)	0.301
	*R* ^2^	0.724

**Fig. 8:**
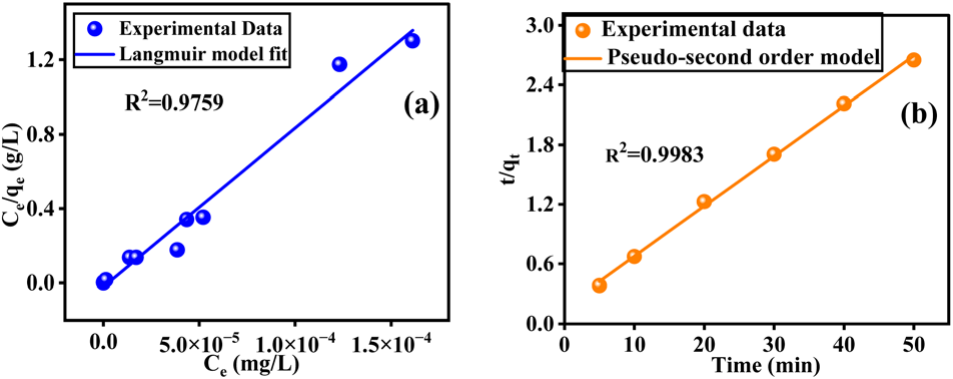
Adsorption isotherm models (a) Langmuir adsorption isotherm model, and kinetic models (b) Pseudo-second order model.

The Langmuir model's linear form is expressed as:5
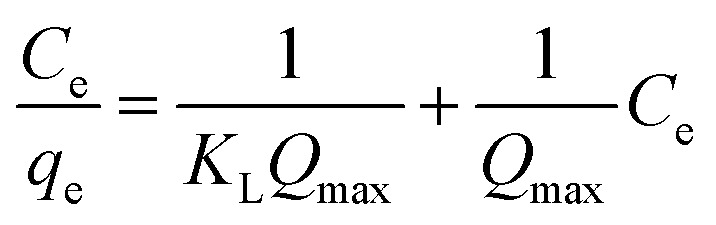
where *C*_e_ (mg L^−1^) is the equilibrium adsorbate concentration, *q*_e_ (mg g^−1^) is the amount adsorbed at equilibrium, *K*_L_ (L mg^−1^) is the Langmuir constant related to adsorption affinity, and *q*_max_ (mg g^−1^) is the maximum adsorption capacity. The model fitting (Fig. 8a) yielded a high coefficient of determination (*R*^2^ = 0.976), indicating an excellent fit. The separation factor *R*_L_, defined by6
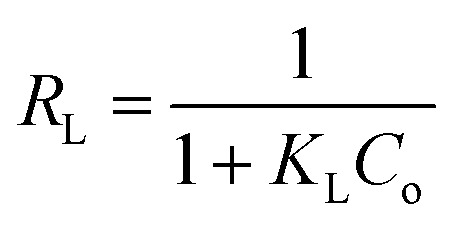
where *C*_o_ is the initial adsorbate concentration, indicating favorable adsorption with *R*_L_ = 0.04257. The Langmuir adsorption model assumes monolayer coverage on a homogeneous adsorbent surface with no lateral interactions among adsorbed molecules.

The characteristics of the adsorption isotherm offer important information about how dye molecules and the adsorbent surface interact. The Langmuir constant (*K*_L_) shows the affinity between the adsorbent and the adsorbate, whereas the maximum adsorption capacity (*q*_max_) represents the monolayer adsorption capacity in the Langmuir model. The adsorption capacity and adsorption intensity in the Freundlich model are described by the parameters *K*_f_ and *n*, respectively, which represent the heterogeneity of the adsorbent surface. In the Dubinin–Radushkevich model, the constant *K*_d_ is related to the adsorption energy, while the mean free energy (*E*) indicates the nature of adsorption—whether physical or chemical. These qualities help in understanding the adsorption process and evaluating how well the developed adsorbent removes dye.

## 5. Kinetic models

The rate of a chemical reaction is governed by the prevailing experimental conditions, as elucidated in chemical kinetics studies. To characterize the adsorption dynamics and establish equilibrium times for Saf-O uptake onto OPP-1 adsorbent, multiple kinetic models were applied, including pseudo-first-order, pseudo-second-order, intraparticle diffusion, and liquid film diffusion models. These models were fitted to the experimental data to determine the relevant kinetic parameters and assess their suitability for describing the underlying adsorption mechanisms. Model fitting enables differentiation between rate-limiting steps, such as surface adsorption, chemisorption, and internal diffusion, and guides optimization of operational parameters for efficient removal performance.^59,60^ The pseudo-second-order kinetic model exhibited the best fit to the experimental data, while the remaining kinetic models are provided in the SI in Fig. S5a–c, and the values are provided in Table 2.

**Table 2:** Kinetic models of OPP-1

Kinetic model	Parameter	Saf-O
Pseudo-first order	*q*_e_ (cal) (mg g^−1^)	144.24
	*k*_1_ (min^−1^)	0.249
	*R* ^2^	0.589
Pseudo-second order	*q*_e_ (cal) (mg g^−1^)	19.841
	*k*_2_ (g mg^−1^ min^−1^)	0.014
	*R* ^2^	0.998
Intra-particle diffusion	*K*_id_ (min^0.5^)	1.168
	*R* ^2^	0.977
Liquid film model	*K*_fd_ (min^−1^)	0.249
	*R* ^2^	0.589

The linear form of the pseudo-second-order kinetic model is mathematically represented by equation.7
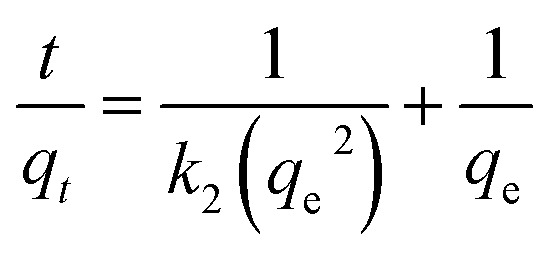
where, *k*_2_ (g mg^−1^ min^−1^) as the rate constant. Plots of *t*/*q*_*t*_ against *t* (Fig. 8b) demonstrated excellent linearity with *R*^2^ > 0.998, and calculated and experimental *q*_e_ values closely matched, confirming that pseudo-second-order kinetics better represent the adsorption process.

## 6. Adsorption of industrial wastewater dyes using synthesized nanocomposite adsorbent OPP-1

**Fig. 9:**
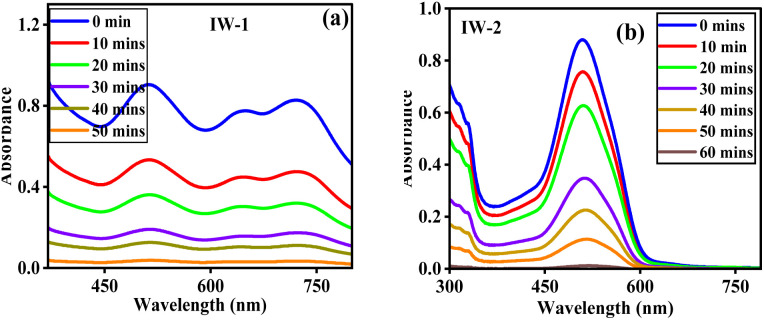
UV-vis spectra of (a) Industrial Wastewater 1 and (b) Industrial Wastewater 2, demonstrating synthetic nanocomposite OPP-1 with a removal efficiency of 95.8% (50 min) and 98.75% (60 min), respectively.

Two industrial wastewater samples, Industrial Wastewater-1 (IW-1) and Industrial Wastewater-2 (IW-2), were used to evaluate the synthesized nanocomposite's performance in practical applications. Time-dependent UV-visible spectroscopy was used to see the adsorption behavior by monitoring changes in absorbance at the characteristic wavelengths of 510 nm and 512 nm, as indicated by the corresponding spectra in Fig. 9a and b. The effective adsorption of the dye by the produced nanocomposite was confirmed by the consistent decrease in absorbance intensity reported for both wastewater samples as the contact time increased. A gradual and significant decrease in absorbance was observed at 512 nm for IW-1 with increasing contact duration, indicating a progressive increase in dye uptake. The removal efficiency increased from 41.04% at the initial stage to 60.07% and 78.98% with extended contact time, subsequently improving to 86.06%, ultimately reaching 95.80% removal in 50 min. The significant affinity of the nanocomposite for the dye, even in the presence of competing contaminants commonly seen in industrial wastewater, is shown by the steady decrease in absorbance intensity. A similar time-dependent adsorption behavior was noted for IW-2 when it was examined at 510 nm. The removal efficiency increased from 28.75% at the initial stage to 60.57% and 74.55% with extended contact time, further increasing to 87.27% and ultimately achieving a maximum removal efficiency of 98.75% in 60 min. The consistent decrease in absorbance intensity indicates the successful removal of the dye molecules from the wastewater and their significant interaction with the active surface sites of the nanocomposite. The adsorption results for both IW-1 and IW-2 indicate the synthesized nanocomposite's effectiveness in dye removal within industrial wastewater systems. High decolorization efficiencies of over 95% were achieved without the need for any additional reducing or oxidizing agents, highlighting the great potential of the discovered adsorbent for real-world industrial wastewater treatment applications.

## 7. Adsorption mechanism

**Fig. 10:**
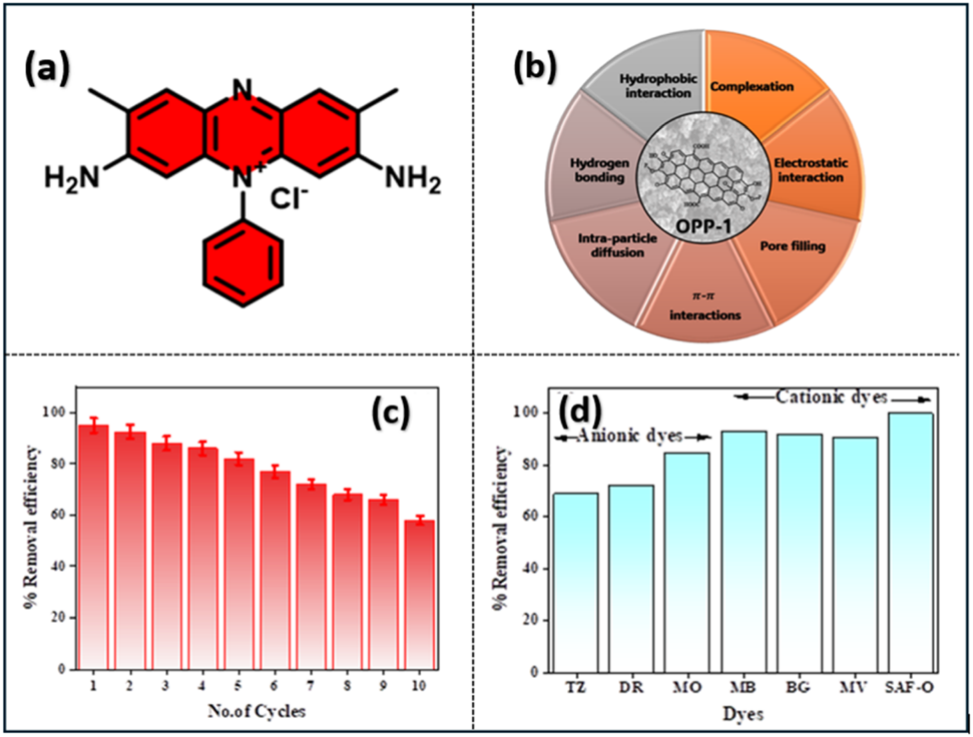
(a) Structure of safranine-O, (b) possible adsorption mechanism for the adsorption of Saf-O by OPP-1 adsorbent, (c) reusability of OPP-1 adsorbent for the removal of Saf-O, (d) OPP-1 adsorbent selectivity towards different cationic and anionic dyes.

The adsorption of Saf-O on OPP-1 proceeds initially *via* rapid external diffusion through the boundary layer, followed by intraparticle diffusion into the porous structure, ultimately reaching equilibrium after a defined contact time. The porosity imparted by H_3_PO_4_ activation facilitates effective pore filling and provides numerous accessible adsorption sites. Strong electrostatic interactions occur between the cationic dye and negatively charged oxygenated surface groups (–OH, –C=O, –C–O–P), particularly at the optimal pH of 10, where the adsorbent surface attains a net negative charge (pH_PZC_ = 6.25). Additionally, π–π stacking between the conjugated carbon aromatic structures and Saf-O's aromatic rings as the structure of Saf-O is shown in Fig. 10a, strengthens surface binding. Complementary mechanisms such as complexation *via* phosphate and carboxyl groups and hydrogen bonding further support chemisorption pathways. Hydrophobic interactions between dye molecules and carbonaceous domains also contribute synergistically. Collectively, kinetic and surface analyses confirm a multifaceted adsorption mechanism, dominated by chemisorption through cooperative interactions including electrostatic attraction, pore filling, hydrogen bonding, π–π stacking, hydrophobic interaction, complexation, and intraparticle diffusion^61^ (Fig. 10b).

## 8. Regeneration studies

Desorption tests were conducted using ethanol as an effective desorbing agent to evaluate the reusability of the synthesized activated carbon. About 5.0 g of dye-loaded OPP-1 was dissolved in 100 mL of 99.5% pure ethanol, resulting in a solid–liquid ratio of 1 : 20 (w/v). To encourage the desorption of the adsorbed dye molecules, the suspension was shaken at 150 rpm for 50 minutes at 50 °C. After treatment, the adsorbent was filtered out and rinsed three times with 50 mL of deionized water each time to get rid of any remaining ethanol and loosely attached dye. After that, the material was dried in a hot-air oven at 60 °C for 12 hours. The regenerated adsorbent demonstrated excellent reusability, maintaining over 82% removal efficiency through the first five cycles (Fig. 10c). A gradual decline was observed in subsequent cycles, with efficiency reducing to approximately 58% by the tenth cycle. This decrease likely arises from partial pore blockage, loss of active functional sites, or irreversible dye binding, phenomena commonly encountered in activated carbon regeneration. Despite this decline, the material's stability and regeneration capacity affirm its practical potential for multiple adsorption–desorption cycles in wastewater treatment applications, aligned with established regeneration strategies including solvent extraction and thermal methods.

## 9. Selectivity of dyes

The selectivity of OPP-1 toward various dyes was assessed by examining the adsorption capacities for cationic dyes such as MB, MV, Saf-O, and anionic dyes such as Tartrazine (TZ), Direct Red (DR), and Methyl Orange (MO). Results demonstrate that OPP1 exhibits a markedly higher affinity for positively charged dyes, with removal efficiencies exceeding 90% for MB (93.3%), BG (91.8%), MV (90.9%), and Saf-O (99.99%), as shown in Fig. 10d. Conversely, the adsorption efficiencies for anionic dyes such as TZ (68.9%), DR (72.4%), and MO (85.0%) were significantly lower. This preferential adsorption is primarily driven by electrostatic interactions, as the surface of OPP1 is predominantly negatively charged at the experimental pH (pH 10), facilitating strong electrostatic attraction with cationic dyes. The reduced affinity for anionic dyes stems from electrostatic repulsion, limiting their adsorption. Surface functional groups, such as hydroxyl and carboxyl groups, further enhance active site interactions with cationic dyes, contributing to the high selectivity observed. These findings affirm OPP-1's potential as a selective adsorbent for the removal of cationic dyes in wastewater treatment processes.

## 10. Phytotoxicity studies

**Fig. 11:**
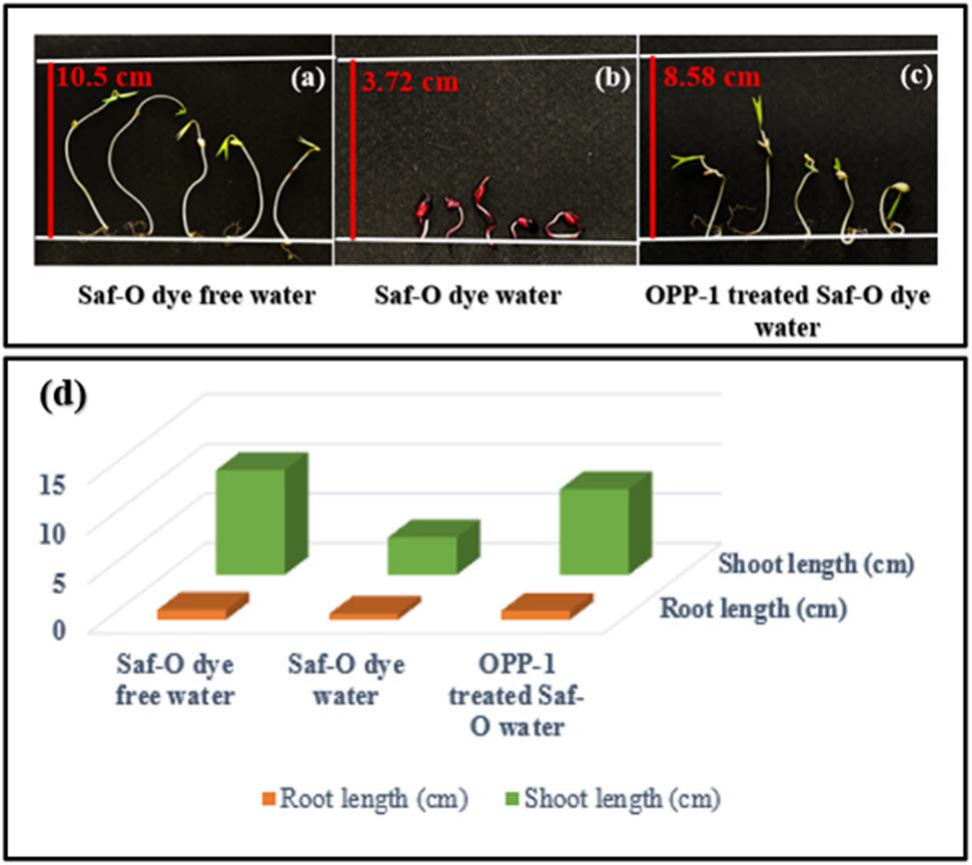
Phytotoxicity test of *Vigna radiata* seeds irrigated with (a) Saf-O dye free water, (b) Saf-O dye water, and (c) OPP-1 treated Saf-O water, (d) bar diagram showing seed germination growth in various water samples with respect to root and shoot lengths.

Fig. 11a–c presents the phytotoxicity assessment of *Vigna radiata* seeds subjected to (a) Saf-O dye free water, (b) Saf-O dye water, and (c) OPP-1 treated Saf-O dye water, to evaluate the environmental safety of the treated effluent. Seeds germinated in dye-free water demonstrated 100% germination, with rapid development indicated by mean shoot and root lengths of 10.50 ± 0.15 cm and 1.0 ± 0.16 cm, respectively, which indicates a non-toxic environment. The untreated dye caused severe phytotoxicity, as evidenced by curled, reddish seedlings and only 60% germination in seeds irrigated with the Saf-O dye solution (100 mg L^−1^). The seeds also showed a significant decrease in root and shoot lengths. It's interesting to note that seeds cultivated in water treated with OPP-1 adsorbent showed significant recovery, reaching 80% germination with mean shoot and root lengths of 8.58 ± 0.27 cm and 0.9 ± 0.31 cm, respectively, as shown in the bar diagram in Fig. 11d, and the table is shown in Table S2. Phytotoxicity assays using *Vigna radiata* revealed that OPP-1-treated safranin-O solutions restored root and shoot growth to approximately 91% and 82% of the negative control, respectively, indicating significant recovery relative to the pronounced growth inhibition observed in the positive control. This recovery demonstrates that the OPP-1 adsorbent was effective in degrading Saf-O. The enhanced germination rate and elongation of seedlings in the treated sample reveal that the post-treatment water is less phytotoxic, indicating that it may be suitable for safe discharge or agricultural reuse without having an adverse effect on the environment.

## 11. Comparison studies

Table 3 compares adsorption capacities, equilibrium times, and recyclability of various adsorbents for Saf-O removal. Functionalized and porous materials consistently exhibit enhanced adsorption capacities and faster kinetics due to improved electrostatic and π–π interactions. Regeneration approaches, such as solvent washing or thermal treatment, critically impact the long-term usability of adsorbents. Within this context, OPP-1 demonstrates excellent adsorption capacity, rapid adsorption kinetics, and durable recyclability, underscoring its potential as a viable adsorbent for efficient dye removal in aqueous environments.

**Table 3:** Comparison table of various materials and their adsorption capacity

S. no.	Material	Adsorption capacity (mg g^−1^)	Recyclability	Time (min)	Ref.
**Saf-O dye**					
1	MgO decked multi-layered graphene	∼127.3	3 cycles	120	62
2	CuO-NP	189.54	4 cycles	480	63
3	Alginate/pomegranate peels beads	30.769	7 cycles	30–180	64
4	Magnetite/Ag nanocomposite	46.3 (Saf-O), 38.46 + 34.97 (binary Saf-O + MB)	5 cycles	90	65
5	SDS-coated Fe_3_O_4_ nanoparticles	769.23	6 cycles	10	66
6	Ferric@ nanocellulose/nanohydroxyapatite bio-composite	239.23	5 cycles	1440 (24 h)	67
7	Ferruginous kaolinite	59.3	5 cycles	420	68
8	Leo-Ca-Alg beads	0.55	10 cycles	120	69
9	Nanomagnetite/copper oxide/potassium carrageenan nanocomposite	384.61	5 cycles	1440 (24 h adsorption), 10 (photo-Fenton)	70
10	Nanocellulose from coconut coir	∼83	6 cycles	270	61

**MB dye**					
11	Banana stem	101.01	4 cycles	90 min	71
12	Acacia mangium wood	159.89	—	—	72
13	Banana leaves	48.01		20 min	73
14	Orthophosphoric acid-treated pomegranate peels	14.03	3 cycles	120 min	54
15	H_3_PO_4_-activated chicken bone adsorbent	49.56	3 cycles	150 min	74

**BG dye**					
16	Carboxymethyl cellulose and sodium alginate crosslinked by epichlorohydrin	864.8	4 cycles	360, 480	75
17	Activated carbon derived from guava seeds	80.5	—	30	76
18	Corn cob activated carbon	238.09	4 cycles	60 min	77
19	Activated carbon derived from date pits	77.8	—	55	78
20	Chemically treated Lawsonia inermis seed powder	34.96	5 cycles	180	79

**MV dye**					
21	Phragmites australis activated carbon	147.02	—	45 and 150 min	80
22	Raw date seeds	59.5	3 cycles	60 min	81
23	Yellow passion fruit peel	485.4	5 cycles	20 min	82
24	Modified rice husk	154.49	5 cycles	30 min	83
25	Biomaterial prepared from Zizyphus Spina-Christi seed	476.19	—	30 min	84

## 12. Conclusion

This study illustrates the successful transformation of OP, an abundant agricultural waste, into a high-performance activated carbon (OPP-1) *via* H_3_PO_4_ activation and pyrolysis at 600 °C. The engineered OPP-1 exhibits a high specific surface area (535.5 m^2^ g^−1^), well-defined mesoporosity, and a hydrophilic surface enriched with heteroatoms (O, N, P), which collectively enhance its adsorption capabilities. Structural characterizations, including Raman spectroscopy, SEM, TEM, and XPS, confirmed enhanced graphitization, diverse porous morphologies, and abundant functional groups that facilitate dye interaction. Thermal analyses further demonstrated OPP-1's stability up to 600 °C, highlighting its suitability for practical application. OPP-1 exhibited strong selectivity toward cationic dyes, achieving removals of Saf-O (99.99%), MB (93.3%), BG (91.8%), and MV (90.9%), while anionic dyes showed lower uptake. Based on this selectivity, safranin-O was chosen as the model dye for detailed investigation. The adsorption behavior aligns well with the Langmuir isotherm and pseudo-second-order kinetic models, indicative of monolayer chemisorption governed by synergistic mechanisms such as electrostatic attraction, π–π stacking, hydrogen bonding, pore filling, and intraparticle diffusion. The practical applicability of the synthesized nanocomposite was evaluated using two real textile effluents (IW-1 and IW-2) by time-dependent UV-visible spectroscopy. A continuous decrease in absorbance at 512 nm (IW-1) and 510 nm (IW-2) confirmed effective dye adsorption. The removal efficiency reached 95.80% for IW-1 within 50 min and 98.75% for IW-2 within 60 min, even in the presence of coexisting contaminants. Phytotoxicity assays with *Vigna radiata* showed that OPP-1-treated safranin-O solutions restored root and shoot growth to about 90% and 82% of the negative control, respectively, showing significant recovery compared to the severe inhibition observed in the positive control. Significantly, OPP-1 demonstrated excellent regeneration performance, maintaining high adsorption efficiency over ten cycles using ethanol as a regenerant. This research not only valorizes agro-waste into a sustainable, efficient dye adsorbent but also provides a scalable route to address water pollution. The combined insights into structure–function relationships, adsorption mechanisms, and operational durability establish OPP-1 as a promising candidate for sustainable wastewater treatment, particularly in removing hazardous cationic dyes.

## Author contributions

M. Bhavani Lakshmi: responsible for the initial draft, methodology development, comprehensive review, and final editing of the manuscript. Alibasha Akbar: engaged in the review process and editing, ensuring clarity and coherence throughout the document. Paramita Pattanayak, Tanmay Chatterjee, Archana V.: conducted formal analysis, reviewing and editing the text for technical accuracy. Mihir Ghosh: provided oversight and conceptual framework, alongside contributions to writing, review, and editing processes, ensuring the integrity of the research narrative.

## Conflicts of interest

There are no conflicts to declare.

## Supplementary Material

RA-016-D6RA00482B-s001

## Data Availability

The findings of this study are supported by data and can be obtained from the corresponding authors upon reasonable request. Supplementary information: surface chemistry, adsorption isotherm/kinetic models, and phytotoxicity data. See DOI: https://doi.org/10.1039/d6ra00482b.
