# Supplementary material for: Greener synthesized P-doped carbon for dual applications: selective cationic dye removal with phytotoxicity assessment and industrial effluent treatment
Source: RSC Adv. 2026 Mar 31;16(19):17507–21. doi: 10.1039/d6ra00482b (PMC13037382; doi:10.1039/d6ra00482b)
Supplement: RA-016-D6RA00482B-s001 [file RA-016-D6RA00482B-s001.pdf]

Supplementary Information (SI) for RSC Advances.
This journal is © The Royal Society of Chemistry 2026

Supplementary information

Greener synthesized P-doped carbon for dual applications: Selective cationic dye removal with phytotoxicity assessment and industrial effluent treatment

M. Bhavani Lakshmi [a], Alibasha Akbar [a], Paramita Pattanayak[b], Tanmay Chatterjee [b], Archana V [c], Mihir Ghosh [a*]

[a] Department of Chemistry, SRM Institute of Science and Technology, Kattankulathur 603203, Tamil Nadu, India
[b]Department of Chemistry, Birla Institute of Technology and Science, Pilani – Hyderabad Campus, Jawahar Nagar, Kapra Mandal, Hyderabad, Telangana 500078, India
[c]CAMRIE, School of Physics, Indian Institute of Science Education and Research Thiruvananthapuram (IISER TVM), Maruthamala P.O., Vithura, Thiruvananthapuram, Kerala 695551, India
**Corresponding author E-mail address: mihirg@srmist.edu.in*

1. Methods:

1.1 Acid/base titrations (Boehm's titration)

The surface characteristics (acidic or basic) of the nanohybrid materials were evaluated using Boehm's selective acid/base neutralization method.

The number of acidic groups on the activated carbon surface was determined using the Boehm titration method. A 0.1 M NaOH solution (50 mL) was placed in a conical container with 0.1 g of activated carbon sample. The conical bottle was subjected to oscillation in a shaker for 24 h at 25 °C (160 rpm) concurrently with a blank experiment at the same conditions. After oscillation, the solution was filtered, and 10 mL of the filtrate was titrated using HCl (0.1 M) and phenolphthalein as the indicator. Furthermore, the NaOH solution was replaced with $NaHCO_3$ solution (0.1 M) and Na_2CO_3 solution (0.1 M) to repeat the oscillation and filtering procedures. Hydrochloric acid (0.1 M) was used during titration, with bromocresol green-methyl red serving as the indicator. NaOH neutralizes the carboxyl, lactone, and phenolic hydroxyl groups, $NaHCO_3$ neutralizes the carboxyl group, and Na_2CO_3 neutralizes the carboxyl group and lactone group. The number of oxygen-containing functional groups was computed based on the amount of alkali solution consumed during the reaction. The functional groups(mmol/g) were determined using the following formula:

$$Functional\ groups\ (mmol/g) = \frac{(Normallity \times Volume_{consumed})}{Molecular\ weight} * \frac{V_{initial}}{V_{titrated}}$$

N represents the normality of the titrant; $V_{consumed}$ denotes the volume of the titrant consumed; $V_{initial}$ denotes the total volume of the solution; $V_{titrated}$ is the volume taken for titration; and M.W denotes the molecular weight of the titrant.

Table S1: Boehm's titration method

Activated Carbon made from onion peels (OPP-1)	Carboxyl groups	Lactone	Phenolic	Acidity	Basicity
**Adsorbed(mmol/g)**	0.9599	0.0109	0.0439	1.0147	0.125

1.2 Determination of pHzpc:

The pHzpc was determined to examine the surface charge of the activated carbon. The solid-addition technique was used to determine the point of zero charge (pHpzc) of the adsorbent. A 45 mL solution of 0.1 mol L^{-1} NaCl was transferred to a set of 100 mL conical flasks. The initial pH values of the solution were adjusted from 1.0 to 11.0 by the addition of either 0.1 mol L^{-1} HCl or 0.1 mol L^{-1} NaOH solutions. The solution volume in each flask was precisely adjusted to 50 mL by adding NaCl solution. Subsequently, 0.1 g) was added to each flask, and the mixtures were stirred at 150 rpm. The final pH values (pH_f) of the solutions were determined after 48 h. The difference between the initial and final pH values ($\Delta pH = pH_i - pH_f$) was plotted against pH. The pHpzc was determined at the point where the resultant curve intersected the abscissa, when $\Delta pH = 0$.

1.3 Zeta potential vs pH

The zeta potential of OPP-1 in pure water was assessed using a zeta potential analyzer (Zetasizer, Malvern Instruments, Worcestershire, UK, model Nano ZS) at different pH levels. The activated carbon suspension was diluted with water and sonicated for 30 min in an ultrasonic bath. The USC-1400 model operated at a distinct frequency of 40 kHz within the ultrasound spectrum. A Malvern 3000 Zetasizer NanoZS (Malvern Instruments, Malvern, UK) was used to measure the average particle size of the activated carbon. Particle diffusion under Brownian motion was measured, and the data were subsequently translated into particle size and size distribution using dynamic light scattering. It is employed in laser Doppler microelectrophoresis to create an electric field for the dispersion of the activated carbon suspension.

1.4 Freundlich adsorption isotherm:

The Freundlich model, describing multilayer adsorption on heterogeneous surfaces, is represented by:

$$\ln q_e = \ln K_f + \frac{1}{n}\ln C_e \tag{1}$$

where K_f and n Freundlich constants are indicative of adsorption capacity and intensity. From the plot of $\log q_e$ versus $\log C_e$, the values of $n = 5.7$ and K_f (Table 1) confirm favorable and intensive adsorption $(n > 1)$ as shown in Figure S4a.

1.5 D-R adsorption isotherm:

The Dubinin–Radushkevich (D–R) isotherm (Figure S4b), based on a Gaussian energy distribution, provides insight into the adsorption nature by estimating the mean free energy E

$$E = \frac{1}{\sqrt{2K_d}} \tag{2}$$

where K_d ($mol^2\ kJ^{-2}$) is the D–R constant. The parameters and R^2 values are summarized in Table 1.

1.6 Pseudo-first order model:

The Lagergren equation is one of the earliest known formulations that describes the rate of adsorption in liquid-phase systems. It is expressed as:

$$log(q_e - q_t) = log q_e - \frac{k_1 t}{2.303} \tag{3}$$

where q_t and q_e (mg/g) are the adsorption capacities at time t and at equilibrium, respectively, and k_1 (min⁻¹) is the rate constant. Linear plots of $ln(q_e - q_t)$ versus t (Figure S5a) yielded low correlation coefficients (R^2), indicating poor fit and suggesting that the pseudo-first-order model may not adequately describe Safranine-O adsorption on OPP-1.

1.7 Intraparticle diffusion model:

The intraparticle diffusion model, as proposed by Weber and Morris, evaluates the diffusion rate of adsorbate molecules within the pores of the adsorbent. It is expressed by:

$$q_t = k_i \times t^{0.5} + I \qquad (4)$$

where q_t is the adsorption capacity at time t, k_i is the intraparticle diffusion rate constant (mg g^{-1} $min^{-0.5}$), and C represents boundary layer thickness. The rate constant k_i is obtained from the slope of q_t versus $t^{0.5}$ plots (Figure S5b), with corresponding values summarized in Table 3. A correlation coefficient $R^2 = 0.978$ indicates that while intraparticle diffusion also significantly contributes to the adsorption kinetics, it is not the major rate-limiting step. But the pseudo-second order kinetics plays a major portion in the adsorption, as well as intraparticle diffusion, which supports the kinetics in the adsorption of safranin-O. The intercept C implies additional resistance from the external liquid film, revealing that both pore diffusion and boundary layer effects concurrently influence the overall adsorption rate.

1.8 Liquid film model:

The liquid-film diffusion model analyzes the role of film diffusion with the equation:

$$-\ln(1 - F) = -k_{fd} \times t \qquad (5)$$

where $F = q_t/q_e$ is the fractional adsorption and K_{fd} (min⁻¹). The film diffusion coefficient. Despite linearity in plots of $-ln(1 - F)$ versus time (Figure S5c), low correlation coefficients indicate film diffusion does not predominantly control the kinetics.

1.9 Phytotoxicity test

The early seed's development and the development of the first structures are essential processes that regulate seed germination and the initial growth of seedlings. Germination assays were performed using *Vigna radiata* (mung bean) seeds to assess the phytotoxicity of Safranin-O (Saf-O) and the treated water. The toxicity of untreated Saf-O solution, OPP-1-treated Saf-O solution, and the control sample (distilled water) was evaluated to investigate their effects on seed germination and seedling development. The phytotoxicity experiment was conducted according to a standard method [55]. Sterilized Vigna radiata seeds were placed in Petri dishes lined with filter paper and moistened by using distilled water (control), a Saf-O solution (20 mg L^{-1}), or an OPP-1–treated Saf-O solution. Each treatment consisted of 20 seeds (n = 20) placed in Petri dishes lined with filter paper and incubated under controlled laboratory conditions for 6 days. Germination percentage, root length, and shoot length were recorded after the incubation period. Raw germination counts revealed that all seeds germinated in the control group (20/20, 100%), whereas only 12 out of 20 seeds germinated in the untreated Saf-O solution (60%). In contrast, the OPP-1–treated Saf-O solution exhibited a substantially higher germination rate of 80% (16/20), indicating a marked reduction in phytotoxicity after adsorption treatment.

$$\text{Germination rate (\%)} = \frac{No.0f\ seeds\ germinated * 100}{Total\ number\ of\ seeds\ germinated}$$

Table S2: Phytotoxic study of Vigna radiata seeds.

Parameter studied	Saf-O dye water	Saf-O dye water	OPP-1 treated Saf-O water
**Germination (%)**	100	60	80
**Length of root (cm)**	1.0 ± 0.16	0.6 ± 0.22	0.9 ± 0.31
**Length of shoot (cm)**	10.50 ± 0.15	3.72 ± 0.10	8.58 ± 0.27

The mean values were calculated with standard deviation (±) and statistically analyzed using Student's t-test (confidence level > 95%, $p < 0.05$).

Figures:

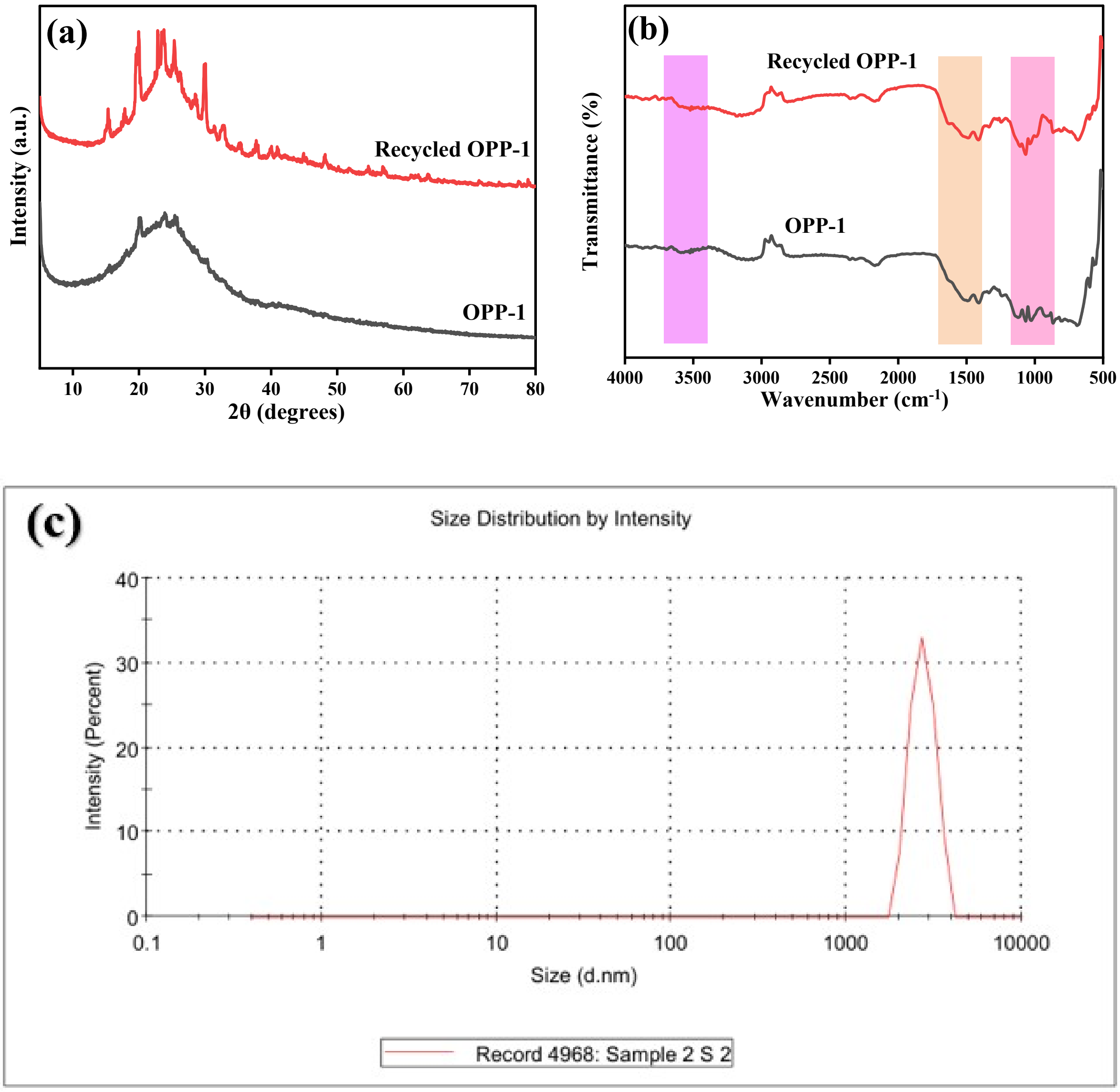

Figure S1: (a) XRD images of OPP-1 and Recycled OPP-1, (b) FT-IR images of OPP-1 and Recycled OPP-1, (c) Particle size distribution of OPP-1 measured by Dynamic Light Scattering (DLS)

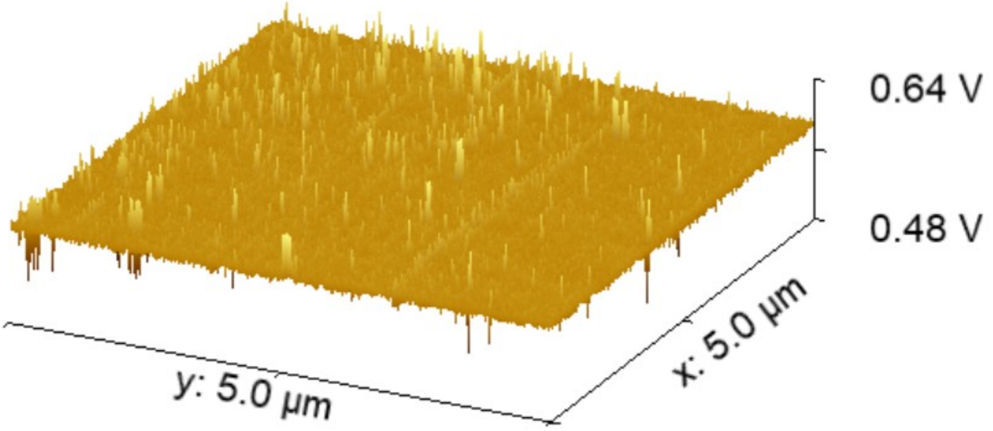

Figure S2: AFM image of OPP-1 adsorbent.

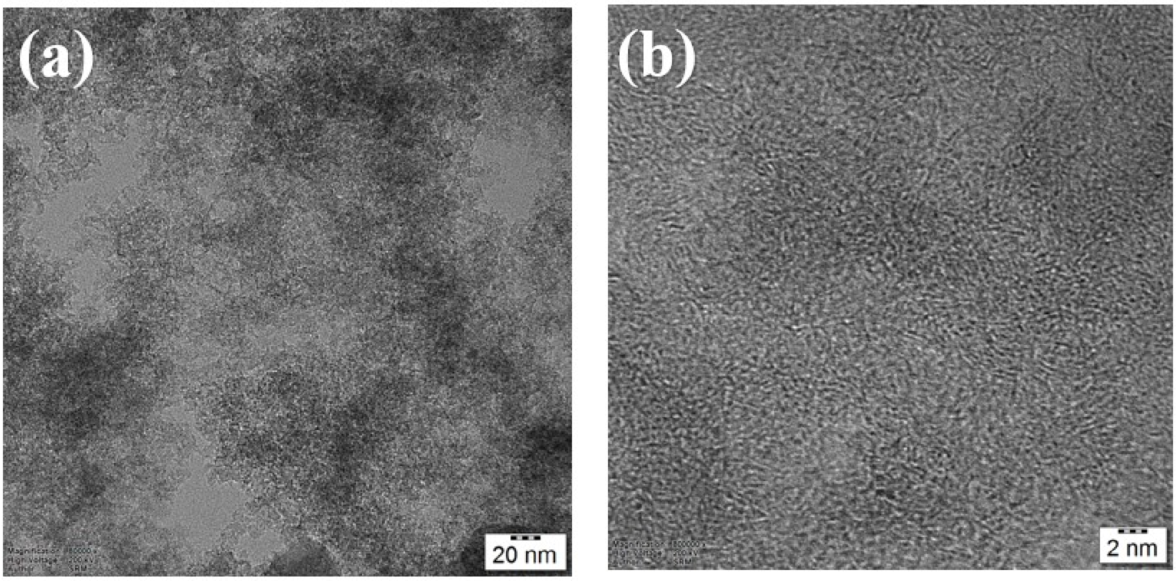

Figure S3: a) Low-resolution TEM image of OPP-1, (b) Lattice fringes of OPP-1.

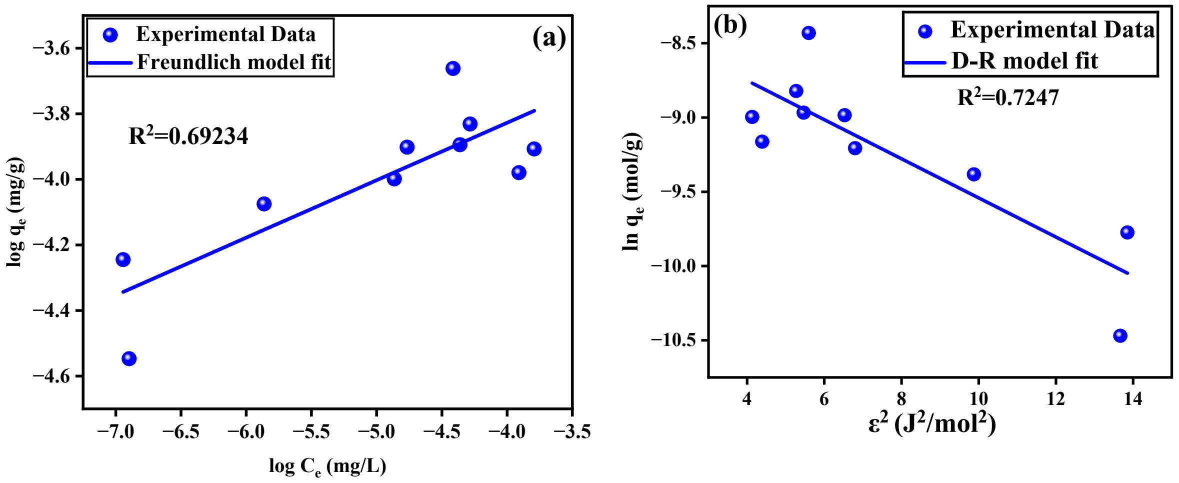

Figure S4: Adsorption isotherm models (a) the Freundlich adsorption isotherm model, (b) D-R adsorption isotherm model.

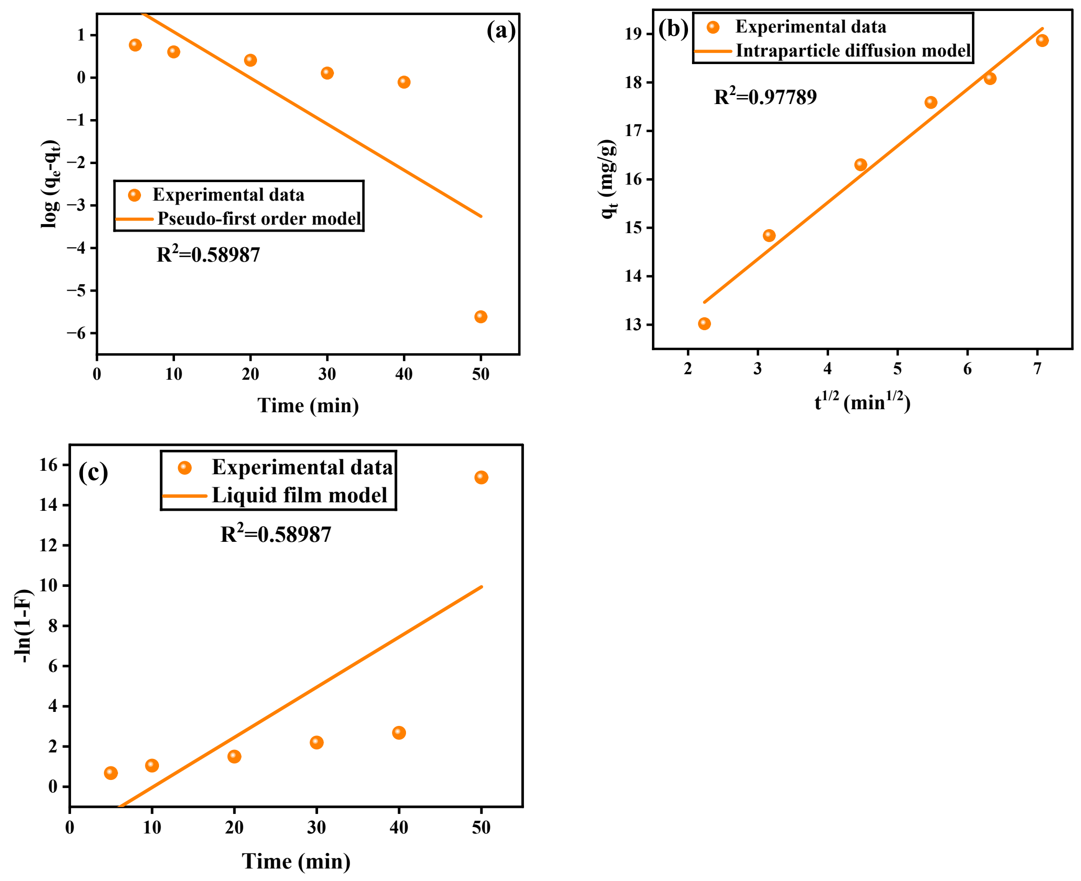

Figure S5: Kinetic models (a) Pseudo-first order model, (b) Intraparticle diffusion model, and (c) Liquid film model.
